# Effects of *Calendula officinalis* extract on liver histopathology, lipid profile, and oxidative stress in rats submitted to a diet rich in cholesterol and carbohydrates

**DOI:** 10.1590/acb383723

**Published:** 2023-10-13

**Authors:** Gleide Gatti Fontes, Rodrigo de Barros Freitas, Palloma Porto Almeida, Luciana Moreira Lima, Silvia Almeida Cardoso, Virginia Ramos Pizziolo, Ricardo Tabach, Almir Gonçalves Wanderley, Ricardo Mario Arida, Afonso Caricati-Neto, Murched Omar Taha, Camilo Amaro de Carvalho, Fernando Sabia Tallo, Francisco Sandro Menezes-Rodrigues

**Affiliations:** 1 Universidade Federal de Juiz de Fora – Juiz de Fora (MG) – Brazil.; 2 Universidade Santo Amaro – School Medicine – São Paulo (SP) – Brazil.; 3 Universidade do Estado do Rio de Janeiro – Rio de Janeiro (RJ) – Brazil.; 4 Universidade Federal do Estado do Rio de Janeiro – Rio de Janeiro (RJ) – Brazil.; 5 Universidade Federal de Viçosa – Viçosa (Minas Gerais) – Brazil.; 6 Universidade Federal de São Paulo – São Paulo (SP) – Brazil.

**Keywords:** Calendula, Dyslipidemias, Flavonoids, Oxidative Stress, Lipids

## Abstract

**Purpose:**

To evaluate the modulatory properties of *Calendula officinalis* L. (Asteraceae) (*C. officinalis*) extract on cafeteria diet-fed rats.

**Methods:**

A cafeteria diet was administered *ad libitum* for 45 days to induce dyslipidemia. Then, the rats were treated with the formulations containing *C. officinalis* in the doses of 50, 100, and 150 mg/kg or only with the vehicle formulation; the control group received a commercial ration.

**Results:**

The cafeteria diet decreased glutathione S-transferase activity and high-density lipoprotein plasmatic levels and damaged the hepatic architecture. The *C. officinalis* extract was able to reduce lipid infiltration in liver tissue and to modulate oxidative stress and lipid profile markers.

**Conclusions:**

The correlations between the variables suggest a pathological connection between oxidative stress markers and serum lipid profile.

## Introduction

The cafeteria diet is a well-established model of a high-fat and or high-sugar diet. It is represented by highly energetic and palatable human foods that induce hyperphagia and weight gain in rodents and mimic obesity and its comorbidities^1^. Obesity is an important risk factor for the evolution of dyslipidemia. This disorder (dyslipidemia) is characterized by increased levels of cholesterol, triglycerides, high-serum low-density lipoproteins (LDL), and low-serum high-density lipoproteins (HDL), and it is the main factor risk to ischemic heart disease^2^. In addition to lifestyle modification, statins are the cornerstone of therapy. More recently, new drugs have been approved as monoclonal antibodies targeting proprotein convertase subtilisin/kexin type 9 for the treatment of hypercholesterolemia, volanesorsen, targeting apo C-III, for the handling of familial chylomicronemia syndrome, as well as bempedoic acid, evinacumab, lomitapide, and inclisiran to reduce LDL-C blood level^3^,^4^. However, these drugs have a high cost and thus are inaccessible to most people.

Ethnomedicinal practices have been used as a source for drug discovery programs in the treatment of several diseases^5^. In the case of drugs that act on the cardiovascular system, statins remain the best example of drugs from natural sources. *Calendula officinalis* L. (Asteraceae) is an annual herb, native to the Mediterranean and cultivated in several countries, including Brazil^6^. Its use in wound healing and as an anti-inflammatory agent^7^ has already been reported in the literature. In addition, the hydroalcoholic extract from its flowers has low toxicity in rodents^8^. Due to its large number of flavonoids, *C. officinalis* presents a lipid-lowering effect^9^, demonstrating therapeutic potential in the control of dyslipidemias and the prevention of cardiovascular diseases.

In this perspective, the development of additional and alternative treatments is still highly necessary for dyslipidemia therapy. In this study, we evaluated the modulatory effect of an herbal formulation containing different doses of *C. officinalis* in the lipid profile and oxidative stress markers of Wistar rats with induced dyslipidemia compared to commercial diet-fed rats. The flavonoid content in the extract was measured, and liver histological changes were also investigated.

## Methods

### Animals and ethics statement

Four-week-old male Wistar rats were obtained from the Animal Center of the Universidade Federal de Viçosa (UFV). The animals went through a 12-day adaptation period receiving a commercial diet and water *ad libitum*. They were randomly separated and housed in polypropylene cages (two animals per cage) and kept under controlled lighting conditions (12-h light/dark cycles) at a controlled temperature (21 ± 2°C). The Ethics Committee of Animal Use of the UFV approved all experimental procedures (protocol 09/2014) and was conducted following the Ethical Principles on Animal Experimentation adopted by the National Council of the Control of Animal Experimentation (CONCEA, No 11.794/2008).

### Plant material, preparation of crude extract, and herbal formulation

Flowers of *C. officinalis* were obtained from an Empresa de Pesquisa Agropecuária de Minas Gerais (EPAMIG) Experimental Farm in Minas Gerais, Brazil. The voucher specimen of the plant was identified by Dr. Eric Koiti Okiyama Hattori, a professor from the Department of Botany at the UFV, and deposited at the UFV herbarium under number (VIC 41899). The flowers were washed in distilled water and air-dried at 40°C. The petals were crushed using a knife mill, and the yield of the powder was calculated. The ethanolic extract contained 100 g of dried petal powder macerated in 500 mL ethanol with 0.3% citric acid (20% m/v). The extract was filtered and concentrated at 40°C for the removal of ethanol, and then lyophilized (Lyophilizer LC1500 – Terroni), at -55°C. The lyophilized extract (10%) was added to a commercial aqueous formula with 20% propylene glycol, 0.4% carboxymethylcellulose, 0.1% methylparaben, 0.05% propylparaben, 0.03% saccharin, and purified water (q.s.p. 100 mL), until complete solubilization.

### Experimental design

Twenty rats were induced, approximately 40 days old, average weight of 120 g, upon arriving at the vivarium, underwent a 12-day adaptation period receiving commercial feed brand Presence and water *ad libitum* and were randomly distributed in cages individual, made of opaque polyethylene material and closed with a stainless steel lid, shaving-type bed, average temperature of 22 °C, kept under controlled lighting conditions (12-hour light/dark cycles).

The animals were induced to dyslipidemia through a cafeteria diet (hyperlipidic) for 45 days (CAF), administered *ad libitum* throughout the experiment, both induction and treatment periods (Table 1). After 45 days, the rats were randomly separated into four distinct groups (n = 5/group). The different groups were treated with the herbal formulation containing *C. officinalis* (50, 100, and 150 mg/kg designated respectively as C50, C100, and C150) or the formulation vehicle (Vei) for 20 days by gavage, once per day^10^. The anti-hyperlipidemic activity *in vivo* of *C. officinalis* at doses of 50 mg/kg and 100 mg/kg was previously reported, with reduction of serum lipids in diabetic rats^11^.

**Table 1:** Composition of the cafeteria diet (g/1000 g diet) and commercial ration (%/1000 g diet).

Cafeteria diet	Quantity (g)	Commercial Ration	Quantity (%)
Ham patê	223.0	Crude Protein	23
French fries	111.0	Ethereal Extract	4.0
Bacon	111.0	Fibrous Matter	5.0
Mortadela	111.0	Mineral Matter	10.0
Sweet biscuit	111.0	Calcium	1.3
Chocolate milk powder	111.0	Phosphorus	0.85

Source: Elaborated by the authors.

The control group (n = 5) received a commercial ration (CR) and was treated with water through gavage so that all animals underwent the same stress. Every three days, body weight and food consumption were recorded. After 65 days of experimentation, the animals underwent euthanasia, being previously anesthetized (inhaled) with 100% isoflurane^12^. The animals were fasted overnight (for about 18 hours) on the last day of experimental model before the induction of anesthesia or the collection of blood samples.

### Characterization of lyophilized Calendula officinalis extract

The lyophilized extract was characterized by high-performance liquid chromatography (HPLC). Briefly, the lyophilized was resuspended in methanol, filtered (0.45 μm), and loaded on a reversed-phase column Shim-pack GVP-ODS guard column (10 × 4.6 mm); Shim-pack VP – ODS (150 × 4.6 mm), equilibrated with 0.5% acetic acid (v/v) and 35% of methanol at a flow rate of 1 mL/min. The mobile phase consisted of increased methanol (50% for 50 min), followed by increasing to 90% of methanol (20 min), and then restored to initial conditions. The absorbance was monitored at 254 nm, and UV-Vis spectra were recorded at wavelengths of 200-400 nm for verification of plant components. The quantification of the total flavonoid content (TFC) was determined using the aluminum chloride colorimetric method^13^. The absorbance was measured in a Biospectro SP-220 spectrophotometer at 415 nm. Quercetin (1–10 μg/mL) was used as a standard for the calibration curve. The TFC was calculated from a calibration curve, and the results were calculated and expressed as mg of quercetin equivalents per g of a petal (dry weight).

### Biochemical and histopathological analysis

After euthanasia, blood, and liver samples were collected. The lipid profile was evaluated by serum quantification of total cholesterol, HDL, LDL, and triglycerides. Livers were washed in 0.9% NaCl solution and placed on a paper filter to remove excess humidity and weight (g). Then, the liver fragments were fixed in 10% v/v buffered formaldehyde (neutralized solution at pH = 7.4) and, 24 hours later, stored in 70° INPM alcohol, dehydrated with ethanol in series of 70, 80, 90, and 100 % w/v for 5 hours each and xylene in two series w/v, also for 5 hours each. Then, the tissues were embedded in paraffin blocks, cut in a microtome to a thickness of 5 μm, deparaffinized in xylene, and rehydrated with a series of washes with alcohols of different degrees. Each histological slide was stained with hematoxylin-eosin.

The photos were obtained using an Olympus CX30 microscope coupled to an Olympus U_CMAD3 digital microcamera (Tokyo, Japan), and the observation was performed at 400x magnification.

For stereological analysis, a test system using one of every 20 sections was used in a standard test area. The liver components (hepatocytes nucleus, sinusoidal capillaries, and blood vessels), as well as lipid droplets, were recorded. We used % lipid droplets; % sinusoidal capillaries; % hepatocyte nuclei; % blood vessels; mean ± standard error of the mean (SEM); CR: commercial ration; CAF: cafeteria diet; Vei: CAF + formulation vehicle: C50: CAF + extract formulation at 50 mg/kg; C100: CAF + extract formulation at 100 mg/kg; and C150: CAF + extract formulation at 150 mg/kg.

Statistically significant differences were determined by the analysis of variance (ANOVA) test (n = 5 animals/group); *p < 0.05, ***p < 0.001 *vs*. CAF; #p < 0.05 *vs*. CR; †p < 0.05 *vs*. Vei. [25, 26]. The volume of each component was calculated using Eq. 1:


V=PP/PT (1)

Where: PP: the number of points located on the interest structure; PT: the total number of points in the histological area^14^.

All stereological analyses were performed using ImageJ software (National Institute of Health).

### Evaluation of oxidative stress

Liver samples were homogenized in ice-cold phosphate buffer saline and centrifuged at 10,000 × g for 10 min. The supernatant was used to analyze catalase (CAT), superoxide dismutase (SOD), and glutathione S-transferase (GST) activities. CAT was assessed by measuring the rate of decomposition of H_2_O_2_ 15. The SOD activity was estimated by the xanthine oxidase method based on the production of hydrogen peroxide and the reduction of nitroblue tetrazolium (NBT)^16^. GST activity was determined by the complexation of the reduced glutathione with 1-chloro-2,4-dinitrobenzene at 340 nm^17^. Lipid peroxidation was assessed by analyzing tissue levels of malondialdehyde (MDA). For this, 100 mg of liver samples were homogenized in phosphate buffer solution (PBS) and incubated with a thiobarbituric acid solution (trichloroacetic acid 15%, thiobarbituric acid 0.375%, and HCl 0.25 M) for 15 min to evaluate the levels of thiobarbituric acid reactive substances (TBARS). TBARS formation was monitored by spectrophotometry at 535 nm^18^.

### Statistical analysis

The power analysis with α = 0.05, power = 0.95, and an expected medium effect size of 0.52 was investigated with G Power software (version 3.1.9.2, G*Power, University of Kiel, Kiel, Germany), resulting in an indicated sample size = 30, to avoid type II error. All statistical analyses were performed in the GraphPad Prism 8.0 statistical software (GraphPad Software Inc., San Diego, CA, United States of America). The variables were analyzed using ANOVA followed by Dunnett’s multiple comparison test to compare all groups against CAF or CR groups. Spearman correlation analysis was used to measure the correlation between the variables. Data were expressed as mean ± SEM, and the differences were considered significant when *p <* 0.05 (significance level 5%).

## Results

### Characterization of Calendula officinalis extract

The HPLC quantification of flavonoid compounds with TFC analysis identified about 4.25 mg/g of flavonoids present in the lyophilized extract. The comparison between retention times and the UV spectrum indicated the presence of rutin (8.606 min), morin (12.959 min), and quercetin (15.941 min), as well as several flavonoid derivatives (Table 2).

**Table 2:** Retention times of reference substances and their respective UV-Vis, obtained via high-performance liquid chromatography.

Substance	Retention time (min)
naringin	8.603
rutin	8.606
hesperidin	9.313
morin	12.959
daidzein	14.245
quercetin	15.941
genistein	17.755
kaempferol	18.064

Source: Elaborated by the authors.

### Body weight and energy intake

**Figure 1:**
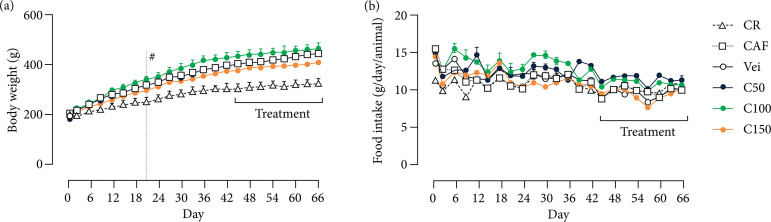
Body weight and food intake from rats submitted to the cafeteria diet and treated with *Calendula officinalis* extract (from 45th day). **(a)** Weight gain; **(b)** average food intake. Statistically significant differences were determined by analysis of variance + Dunnett’s Test (n = 5 animals/group), mean ± standard error of the mean.

Source: Elaborated by the authors. CR: commercial ration; CAF: cafeteria diet; Vei: CAF + formulation vehicle; C50, C100, and C150: CAF + extract formulation at 50, 100 e 150 mg/kg respectively; #*p <* 0.05 all groups *vs*. CR from the 21st day.

All groups started the experiment without significant differences in their body weight. The CAF was able to induce significant weight gain in rats starting from the 21st day of dyslipidemia induction (CAF *vs*. CR, p *<* 0.05). Treatment with *C. officinalis* extract (from the 45th day onwards) did not change the body weight gain profile as shown in Fig. 1a. All groups showed an irregular pattern of food intake (Fig. 1b).

### The effects of Calendula officinalis extract on the liver stereological parameters

The liver weight and stereological parameters are shown in Table 3. The CAF-fed animals had a large liver (p *<* 0.05) than those fed a commercial diet (CR). Administration of *C. officinalis* extract was unable to avoid the increase in liver weight. CAF-fed animals presented damaged hepatic architecture, with a significantly higher percentage (p *<* 0.0001) of lipid droplets in the liver compared to the CR-fed animals. In contrast, the *C. officinalis* extract fully reversed (C50 and C150, p < 0.0001) or partially reversed (C100, p < 0.01) the formation of hepatic droplets induced by CAF-fed animals. The CAF-fed animals showed reduction (p < 0.0001) of sinusoidal capillaries compared to the CR-fed animals. In addition, the *C. officinalis* extract in all doses completely reversed (p < 0.0001) the decreased sinusoidal capillaries. The group that received only the vehicle also induced these same actions.

These data lead us to speculate whether the activity of the extract in the hepatic tissue could be somehow related to the composition of the vehicle. Hepatocyte nuclei and blood vessel parameters were not modified by CAF-fed animals or *C. officinalis* extract group. Furthermore, the extract did not significantly affect the organization of the liver tissue (Fig. 2).

**Table 3:** Weight and stereological parameters of the liver tissue from rats submitted to the cafeteria diet for 45 days and treated orally for 20 days with *Calendula officinalis* extract. Statistically significant differences were determined by analysis of variance + Dunnett’s test (n = 5 animals/group); mean ± standard error of the mean.

	CR	CAF	Vei	C50	C100	C150
Liver (g)	11.14 ± 0.75	14.02 ± 0.33#	12.00 ± 0.43	13.84 ± 0.77	14.32 ± 0.30	12.34 ± 0.84
Lipid Droplets (%)	0.23 ± 0.07	9.09 ± 1.17####	0.75 ± 0.18****	2.79 ± 1.21****	4.40 ± 0.82**	1.63 ± 0.17****
Sinusoidal Capillaries (%)	22.41± 1.24	12.66 ± 0.55####	25.67 ± 1.40****	23.81± 0.77****	24.28 ± 1.00****	24.55 ± 0.41****
Hepatocyte Nuclei (%)	8.69 ± 0.56	7.65 ± 0.44	8.32 ± 0.25	6.28 ± 0.53	6.16 ± 0.34	7.18 ± 0.31
Blood Vessels (%)	1.42 ± 0.39	0.71 ± 0.18	0.66 ± 0.30	1.44 ± 0.41	0.58 ± 0.29	1.86 ± 1.02

Source: Elaborated by the authors. CR: commercial ration; CAF: cafeteria diet; Vei: CAF + formulation vehicle: C50, C100, and C150: CAF + *C. officinalis* extract formulation at 50, 100, and 150 mg/kg;

** p < 0.01,

**** p < 0.0001 *vs*. CAF;

# p < 0.05,

#### p < 0.0001 *vs*. CR.

**Figure 2:**
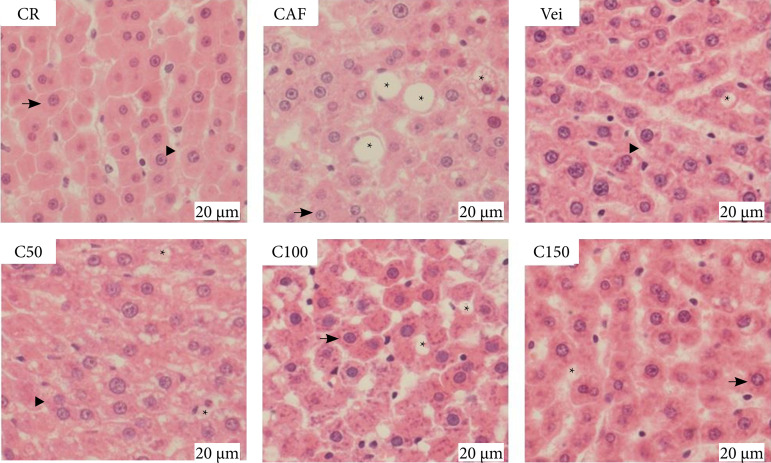
Representative photomicrographs of liver tissue histology in experimental rats fed with a commercial diet or after dyslipidemia induction following treatments (hematoxylin and eosin staining, 400x, bar = 20 μm).

Source: Elaborated by the authors. Triangle: inflammatory infiltrate; Star: lipid droplet; Arrowhead: sinusoidal capillaries; Arrow: hepatocyte; CR: commercial diet; CAF: cafeteria diet; Vei: CAF + formulation vehicle; C50, C100, and C150: CAF + extract formulation at 50, 100 e 150 mg/kg respectively (n = 5 animals/group).

### Serum biochemical parameters

**Figure 3:**
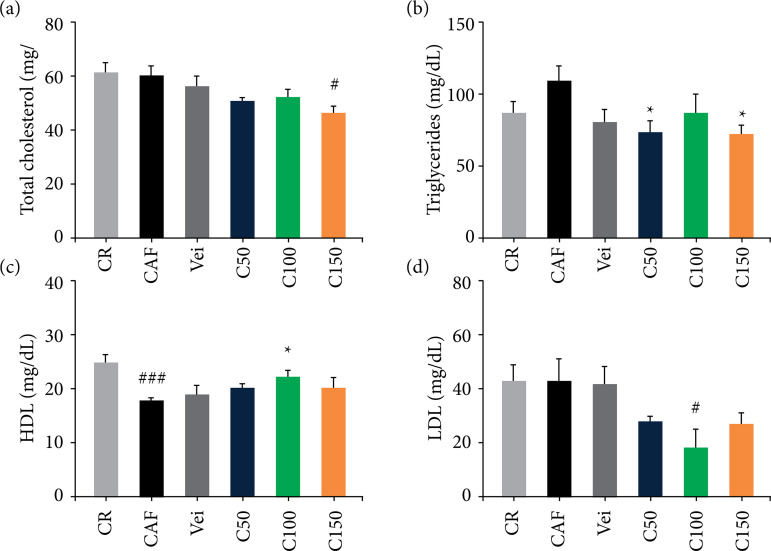
Serum levels of lipid profile between the groups. **(a)** TC; **(b)** triglycerides; **(c)** HDL; **(d)** LDL. Statistically significant differences were determined by analysis of variance + Dunnett’s test (n = 5 animals/group), mean ± standard error of the mean.

Source: Elaborated by the authors. TC: total cholesterol; HDL: high-density lipoprotein; LDL: low-density lipoprotein; CR: commercial diet; CAF: cafeteria diet; Vei: CAF + formulation vehicle; C50, C100, and C150: CAF + extract formulation at 50, 100 e 150 mg/kg respectively; *p *<* 0.05 *vs*. CAF; #p *<* 0.05, ###p *<* 0.001 *vs*. CR.

Regarding biochemical parameters, there was no difference in the total cholesterol (TC) level between CAF *vs*. CR group. However, the *C. officinalis* group C150 significantly reduced (p *<* 0.05) the TC level compared to the CR and CAF groups, as shown in Fig. 3a. There was also no significant difference in the triglyceride level among the CAF *vs*. CR group, but the *C. officinalis* extract decreased (p < 0.05) the triglyceride level in relation to the CAF group (Fig. 3b).

**Figure 4:**
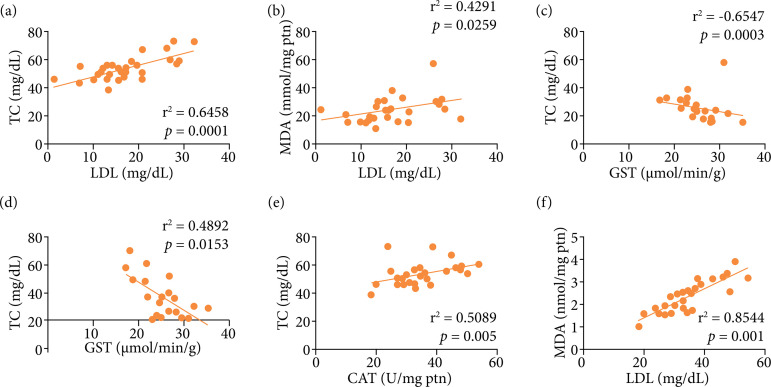
Spearman correlation between serum lipid profile markers and liver oxidative stress markers from cafeteria diet-fed animals treated with *Calendula officinalis* extract and from the control group. **(a)** Correlation between CAT and TC; **(b)** correlation between CAT and MDA; **(c)** correlation between GST and MDA; **(d)** correlation between GST and lipid droplets; **(e)** correlation between LDL and TC; **(f)** correlation between LDL and MDA. Correlations statistically significant were assessed by Spearman correlation analysis, p *<* 0.05.

Source: Elaborated by the authors. CAT: catalase; TC: total cholesterol; MDA: malondialdehyde; GST: glutathione S-transferase; LDL: low-density lipoprotein.

In the cholesterol fractions, there was reduction (p *<* 0.001) in HDL concentration in the group fed the cafeteria diet compared to the group fed with the commercial diet. On the other hand, the HDL level increased (p *<* 0.05) in the C100 group compared to the CAF group was obtained (Fig. 3c). After treatment with *C. officinalis* (C100), there was reduction (*p* < 0.05) in LDL levels compared to the CR group and CAF group (Fig. 3d). There was a strong correlation between LDL and TC levels (r^2^ = 0.6458; p = 0.0001) and MDA (r^2^ = 0.4291; p = 0.0259), as shown in Figs. 4a and 4b.

### Oxidative stress analysis

**Figure 5:**
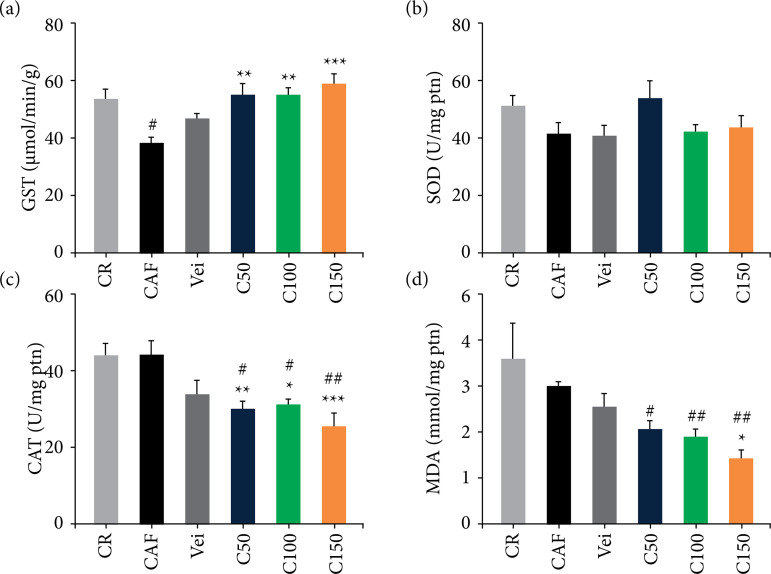
Oxidative stress markers in the liver tissue of cafeteria-diet-fed animals group, and the different treatments. **(a)** GST; **(b)** SOD; **(c)** CAT; **(d)** MDA. Statistically significant differences were determined by analysis of variance + Dunnett’s Test (n = 5 animals/group), mean ± standard error of the mean.

Source: Elaborated by the authors. GST: glutathione S-transferase; SOD: superoxide dismutase; CAT: catalase; MDA: malondialdehyde; CR: commercial diet; CAF: cafeteria diet; Vei: CAF + formulation vehicle; C50: C50, C100, and C150: CAF + extract formulation at 50, 100 e 150 mg/kg respectively; *p < 0.05, **p < 0.01, ***p < 0.001 *vs*. CAF; #p < 0.05, ##p < 0.01 *vs*. CR.

GST activity decreased (p < 0.05) in the cafeteria diet-fed animals compared to commercial diet-fed animals, although the GST activity increased (p *<* 0.01 and p < 0.0001) in the cafeteria diet-fed animals treated with all doses of *C. officinalis* extract, as shown in Fig. 5a. The GST activity showed an inverse correlation with MDA and lipid droplets: the greater the GST activity, the lower the concentration of MDA (r^2^ = -0.6547; p = 0.0003), and the formation of lipid droplets (r^2^ = -0.4892 p = 0.0153), as shown in Figs. 4c and 4d, respectively. No significant changes were observed in the activity of SOD neither in the cafeteria diet nor in the other groups (Fig. 5b).

The CAT activity decreased (p < 0.05) in the animals treated with all doses of *C. officinalis* extract compared to the CR and CAF groups (Fig. 5c). There was a strong positive correlation between CAT activity and TC (r^2^ = 0.5089; p = 0.005), LDL (r^2^ = 0.4498; p = 0.0163, not shown), and MDA (r^2^ = 0.8544; p *<* 0.001), as shown in Figs. 4e and 4f, respectively. Although the MDA production did not differ in CAF *vs*. CR groups, the lipid peroxidation was lower in the C150 group compared to the CAF (p *<* 0.05) and at all doses of *C. officinalis* compared to the CR group (p *<* 0.05 and p *<* 0.01), as noted in Fig. 5c. Additionally, MDA presented correlation with LDL (Fig. 4b) and TC (r^2^ = 0.3886; p = 0.0452, not shown), indicating the role of lipid peroxidation in the serum lipid profile.

## Discussion

We verified the efficacy of a *C. officinalis* formulation in the treatment of CAF diet-fed dyslipidemic rats. The phytochemistry characterization of *C. officinalis* extract identified about 4.25 mg/g of flavonoids present in the lyophilized extract, including morin and quercetin, as well as several flavonoid derivatives. This high flavonoid content is in agreement with the Brazilian legislation for herbal medicines that involves *C. officinalis* formulations, which standardized that total flavonoids should be in the range from 1.6 to 5 mg per 100 g of product^19^. Flavonoids, like morin and quercetin, have already been described as containing protective properties for many biological processes like oxidative stress^20^ and damage caused by lipid accumulation in the liver^21^.

The link between dyslipidemia and oxidative stress has previously been documented in the literature^22^. Herein, we reported in the analysis of oxidative stress and the antioxidant activity of the extract a protective effect against cell damage, that may be explained by the high TFC in the *C. officinalis* extract. There was reduction of GST activity in the cafeteria diet-fed animals compared to untreated animals, possibly due to increased oxidative stress in the hepatic tissue with consequent reduction of antioxidant defenses.

GST has multiple biological functions, mostly related to protecting cells against oxidative stress. It can conjugate GPx to catalyze the reduction of hydrogen peroxide in water^23^. *C. officinalis* extract at all doses completely reversed GST activity in the cafeteria diet-fed animals. Oral administration of flavonoids quercetin was already linked to an increase in GST activity or induction of GST isoforms^23^. Elevated molecular oxygen and an imbalance between increased oxidants and antioxidants can lead to an elevation in radical oxygen species and oxidative damage^24^. Considering the importance of GST antioxidant defense, the negative correlation between its activity and MDA levels shows the major role played by this enzyme and the GST pathway in preventing cell damage.

No significant changes were observed in the activity of SOD neither in the cafeteria diet nor in the other groups, indicating that the cafeteria diet did not induce an increase in the first line of defense against liver tissue oxidative damage^25^. The CAT activity significantly decreased in animals treated with all doses of *C. officinalis.* The decrease in CAT activity can indicate a decrease in free radical production^26^ or less substrate for CAT to decompose due to the detoxification of hydrogen peroxide by the glutathione pathway, indirectly demonstrated in GST activity analysis^27^.

The negative correlation between those variables has been reported in the literature on schizophrenia and diabetes mellitus in the context of sustained cellular stress^27^, possibly preventing the lipid peroxidation process by detoxification of hydrogen peroxide. In our study, we observed a strong correlation between CAT activity and MDA production indicating possible participation of CAT in lipid peroxidation otherwise than preventing. The decreased CAT activity also correlated to LDL and TC reduced levels. The induction of CAT production by oxidized LDL was previously described^27^. Moreover, the investigation of the relation of CAT and serum lipid markers could be investigated further.

Although the MDA production did not differ after the cafeteria, the lipid peroxidation was lower after treatment with all doses of *C. officinalis*. The MDA is an important marker of lipid peroxidation, which is a process associated with the destruction of cell membranes and DNA damage^28^. The reduction of MDA can also be related to the presence of morin^29^ or increased GST activity, which is related to the detoxification of lipid peroxides^30^. We verified a correlation between MDA and LDL and TC levels and between TC and LDL levels. High-fat diets have been linked to the development of atherosclerosis and cardiovascular disease. The modifications of LDL play major roles in the progression of these pathologies^2^,^31^. Previous studies pointed out the importance of MDA-LDL modification in vessel walls and endothelial function in the progression of atherosclerosis^32^–^34^. Furthermore, LDL and MDA individual correlations with TC suggest a pathological connection between TC and rich lipoproteins in atherosclerosis^35^.

High-fat content in the diet can induce accumulation of hepatic cholesterol, hepatic steatosis, and the consequential weight gain of the organ^36^. A cafeteria diet is associated with lipid droplet formation in hepatic tissue. The stereological analysis data partially correlate with the histological analysis. Cafeteria diet induces lipid droplet formation due to triglyceride accumulation in the liver and, herein, the disruption of hepatic architecture, altering hepatocyte nuclei, and sinusoidal capillaries^37^. *Calendula officinalis* extract was effective in reducing the formation of lipid droplets and increasing sinusoid capillaries, indicating a protective action on the liver. Indeed, flavonoids have been described as hepatoprotective biomolecules that decrease hepatic histological damage and inhibit serum and hepatic lipid accumulation^38^. This also contributes to ameliorating oxidative stress, corroborating with the lower production of MDA and the modulation of antioxidant enzymatic activities observed in our analysis.

The cafeteria diet is a high-fat diet with refined carbohydrates, which contributes to dyslipidemia and low-grade inflammation. This intake of a high-lipid diet can generate damage that results in blood lipid disorders^39^. The HDL lipoprotein performs the reverse transport of cholesterol, removing it from peripheral tissues to the liver for excretion through bile and feces^40^.

In this study, the extract was able to restore the HDL level in the dosage of 100 mg/kg. HDL anti-inflammatory and antithrombotic activities confer protection against LDL oxidation in the arterial wall^41^. Data published demonstrate the flavonoids present in the extract used during the experiments are related to enhanced HDL function and increase hepatic serum paraoxonase 1 expression and activity^42^. This protein is a HDL-associated esterase that prevents LDL oxidation^43^ and, therefore, protects the liver and other tissues against oxidative vascular damage.

*Calendula officinalis* has a high flavonoid content, which presents antioxidant and atheroprotective potential. These properties are most likely responsible for the hypolipidemic effect verified in the formulation with *C. officinalis* extract^44^. The data obtained in this study point out that *C. officinalis* extract has a promising use in the treatment and prevention of dyslipidemia and associated diseases, in alignment with previous studies that also indicated the potential medical use of this plant^45^.

In our study, dyslipidemia status was induced through cafeteria diet administration on male Wistar rats and caused alterations in serum lipid profile and oxidative stress markers. The dose of 100 mg/kg produced better results, with a statistically significant reduction of LDL and an increase of HDL in cafeteria diet-fed male Wistar rats compared to those that consumed a commercial diet.

Regarding the modulation of oxidative stress markers and the lipid droplet reduction in liver tissue, different dosages showed distinct modulatory activities. The Spearman correlation between the variables showed the possible main mechanisms that could impair homeostasis leading to pathogenesis, which can be assessed in further investigations. It indicates the use of different dosages or associations for the evaluation of different dyslipidemia phenotypes and variables’ correlation behavior.

## Conclusion

The *C. officinalis* extract was able to reduce lipid infiltration in liver tissue and modulate oxidative stress and lipid profile markers. The correlations between the variables suggest a pathological connection between oxidative stress markers and serum lipid profile. The findings of this study must be seen in light of some limitations: the expression of genes related to lipid profile were not investigated at the transcriptional or protein level; the model used was an animal model; and further studies are needed to extrapolate the effects to humans. These questions could be addressed in future investigations from our group.

## Data Availability

The data will be available upon request.
